# Primary splenic angiosarcoma: a case series of a rare oncological entity and diagnostic challenge

**DOI:** 10.2340/1651-226X.2024.35412

**Published:** 2024-04-15

**Authors:** Iris Dirven, Philippe Leclercq, Lionel D’Hondt, Valentine Delmotte, Pierre Lefesvre, Hendrik Reynaert, Frederik Vandenbroucke, Magali Surmont

**Affiliations:** a Department of Gastro-enterology, Universitair Ziekenhuis Brussel (UZ Brussel), Brussels, Belgium; b Department of Gastro-enterology, CHC MontLégia, Liège, Belgium; c Department of Oncology, CHU UCL Namur site Godinne, Yvoir, Belgium; d Department of Pathology, Universitair Ziekenhuis Brussel (UZ Brussel), Brussels, Belgium; e Department of Radiology, Universitair Ziekenhuis Brussel (UZ Brussel), Brussels, Belgium

**Keywords:** Angiosarcoma, liver failure, primary splenic angiosarcoma, splenic imaging, splenic neoplasms

## Abstract

**Background and purpose:**

Primary angiosarcoma of the spleen (PAS), an exceptionally rare and aggressive neoplasm with high metastatic risk (70%–85%), is frequently diagnosed in an advanced or metastatic stage. It presents diagnostic challenges due to its nonspecific symptomatology and resemblance to benign vascular lesions in various imaging modalities.

**Patients and methods:**

This case series aims to clarify the diagnostic difficulties by comparing imaging characteristics (CT-scan, MRI, and [^18^F]FDG-PET/CT) as well as pathological findings of three PAS cases diagnosed in different stages of the diseases (localized, metastatic, and metastatic with organ failure). Furthermore, a brief review on diagnostic and therapeutic features is included.

**Results and interpretation:**

We suggest [^18^F]FDG-PET/CT as a differentiating tool between benign and malignant splenic lesions and propose a flowchart of a diagnostic algorithm for PAS. For treatment, we advocate for early splenectomy and when systemic therapy is warranted, paclitaxel emerges as a viable first-line option. While it is crucial to acknowledge that further trial data is required to evaluate the efficacy of emerging treatment regimens, designing and conducting trials for PAS is challenging given its scarcity and aggressive behavior. Therefore case reporting remains important.

## Introduction

Malignant splenic tumors are rare, and a primary angiosarcoma of the spleen (PAS) is even more uncommon, constituting less than 1% of all sarcomas and 7.4% of primary malignant splenic tumors [1]. The annual incidence rate is 0.14–0.23 cases/million [2]. There is a slight male predominance and PAS usually develops during the fifth to sixth decade of one’s life. Unlike common lymphoid splenic tumors (e.g. lymphoma), PAS originates from the vascular endothelium, specifically from the endothelial cells lining the splenic sinusoids. This aggressive neoplasm exhibits a high metastatic rate of 70%–85%, primarily involving the liver, lungs, bone, and lymph nodes through hematologic spread [3]. Unfortunately, the prognosis is dismal with a median overall survival (OS) of only 5–6 months [3]. In case of early detection, splenectomy has been reported to increase the median OS to 14 months [2, 4]. Here, we present three cases of PAS, diagnosed at different disease stages in Belgium, along with a discussion on the challenges in clinical, radiographic, and pathological assessment and treatment approaches.

## Case presentations

### Case 1

**Figure 1:**
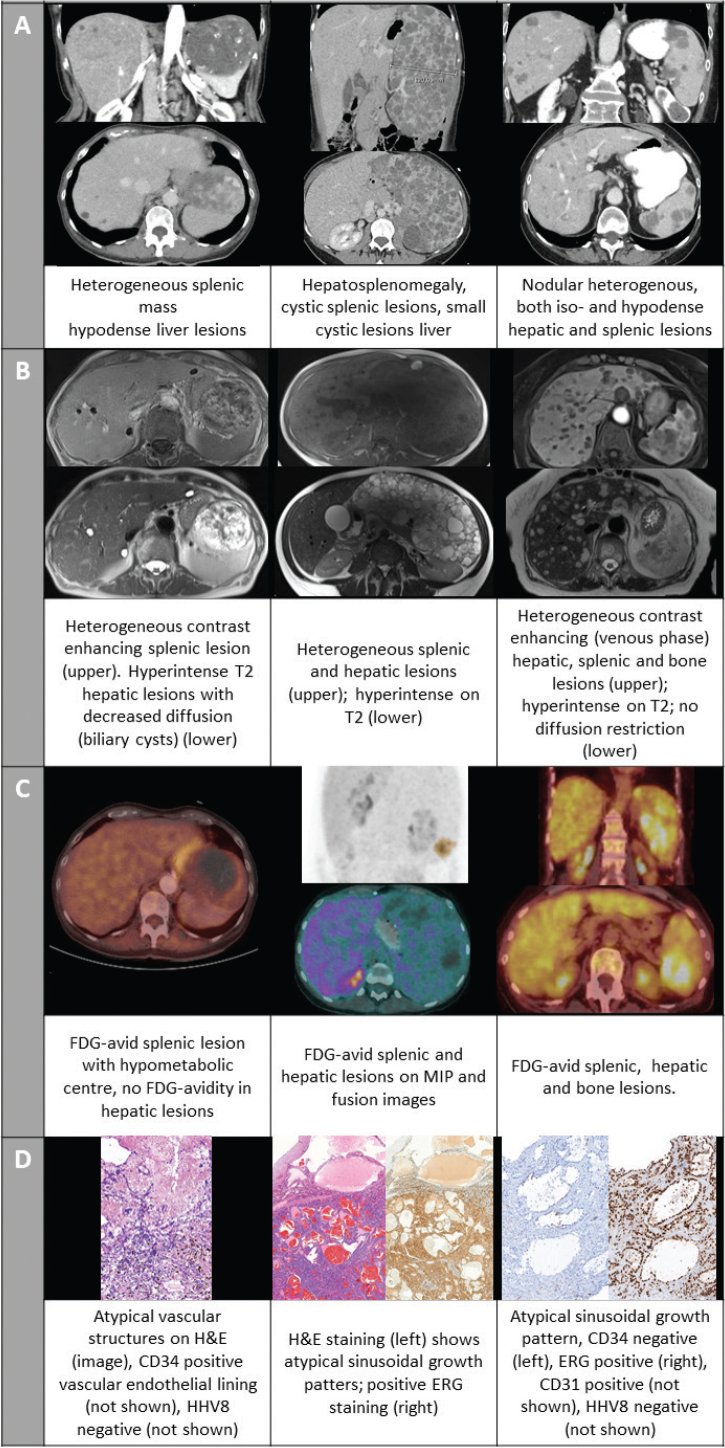
Imaging and pathology slides of cases 1–3. (A) intravenous contrast- enhanced computed tomography (CT) scan; (B) gadolinium-enhanced magnetic resonance imaging (MRI) (C) Fusion images and maximum intensity projection (middle panel only) of 18-fluorodeoxyglucose-positron emission tomography computed tomography ([^18^F]FDG PET/CT); (D) pathology slides with hematoxylin and eosin (H&E) staining and/or immune histochemical staining for vascular markers.

A 70-year-old female, with a medical history of atrial fibrillation and thyroidectomy, was incidentally found to have an abdominal mass during a routine gynecological check-up. She complained only of minor abdominal pain. An abdominal computed tomography (CT-) scan with intravenous (IV) contrast revealed a hypodense heterogeneous splenic mass (97 × 75 mm) and several hypodense infra-centrimetric liver lesions (Figure 1A). Gadolinium-enhanced magnetic resonance imaging (MRI) confirmed the contrast-enhancing splenic lesion, while the lesions in the liver were hyperintense on T2 with decreased diffusion (Figure 1B). An 18-fluorodeoxyglucose-positron emission tomography CT ([^18^F]FDG-PET/CT) showed hypermetabolism at the edges of the splenic lesion, which was absent in the hepatic lesions (Figure 1C). A splenectomy was performed. The histopathological diagnosis confirmed PAS due to the large atypical vascular lesions, positive for the vascular marker CD34 (Figure 1D). Regional lymph nodes were not involved. No adjuvant therapy was initiated, and the patient remained disease-free for 4 years until passing from a non-oncological disease.

### Case 2

A 36-year-old female presented with abdominal pain radiating to the left hypochondria and left flank increasing over a week accompanied with chills without documented fever. Abdominal contrast-enhanced CT-scan revealed splenomegaly (long axis:19 cm) with multiple cystic lesions, as well as hepatomegaly with numerous small cystic lesions (Figure 1A). [^18^F]FDG-PET/CT showed hypermetabolism in both the spleen and liver (Figure 1C). MRI demonstrated heterogeneous and hyperintense T2 regions in both the splenic and hepatic lesions, suggesting lymphangiomatosis in the spleen and angiomata or hamartomatas in the liver (Figure 1B). Tumor markers (carcinoembryonic antigen (CEA), cancer antigen 19.9 (CA 19.9), neuron-specific enolase (NSE)) were within the normal range. Diagnostic splenectomy and liver biopsy confirmed the presence of angiosarcoma in both the spleen and liver with positive staining for the vascular marker ERG (Figure 1D). Due to the metastatic status, the patient initiated systemic treatment with paclitaxel weekly. After several months, the patient was lost to follow-up.

### Case 3

A 75-year-old female with a history of hypertension presented with fatigue and weight loss. Abdominal CT revealed more than 20 hypervascular, contrast-enhancing liver and splenic lesions (Figure 1A). An additional MRI showed T2 hyperintense nodular lesions in the liver and spleen contrast-enhancing in the late-arterial, portal, and venous phase. Similar T2 hyperintense nodular lesions were noted in the spine (Figure 1B). Absence of malignant signs, more specifically no diffusion restriction, was reported. No further investigations were performed at this time. Four months later, a CT-scan and MRI of the lumbar spine were performed for increasing back pain. The bone lesions were isodense on CT and on MRI hypointense on T1 and hyperintense on STIR, suggesting bone metastases. An additional [^18^F]FDG-PET/CT showed hypermetabolic lesions in the liver, spleen, and spine (Figure 1C). A lymphoproliferative disease was ruled out. At initial referral to the gastro-enterology department following the [^18^F]FDG-PET/CT, the patient already exhibited signs of liver failure including ascites and impaired coagulation. No infectious, autoimmune, or metabolic causes were identified; tumor markers alfa-foetoprotein, CA 19-9 and CEA were within the normal range. A liver lesion biopsy showed sinusoidal growth patterns with atypical endothelial cells positive for vascular markers (ERG, CD31) and a high proliferation index Ki-67 (80%). Additional immune histochemical staining was CD34 negative and CD8 positive suggesting a splenic origin and confirming the diagnosis of a liver metastasis of a PAS (Figure 1D). The patient’s liver function deteriorated, impeding the initiation of systemic treatment. She died within 2 weeks of the diagnosis and 7 months after the initial presentation.

## Discussion

The diagnosis and management of a PAS can pose challenges due to its rarity and varied clinical and radiological presentations. We presented three cases diagnosed at different disease stages in Belgium. In the first case, the angiosarcoma was confined to the spleen. The patient remained disease-free during close follow-up after splenectomy without receiving adjuvant treatment. The patients in the second and third case had metastatic disease. However, in the second case the patient remained in good clinical condition with systemic treatment, while the third patient’s liver function quickly deteriorated.

While in most patients PAS is diagnosed in the fifth and sixth decade, it can develop at any age, such as in the 36-year-old patient. The age at diagnosis does not seem to influence survival [1].

The clinical presentation of PAS can range from an asymptomatic incidental finding to nonspecific complaints such as abdominal discomfort, weight loss, fatigue, or anorexia. In some cases, splenic rupture is the presenting symptom, leading to early intra-abdominal seeding and metastasis. On clinical examination, there are no specific cues; in case of large lesions or a metastatic setting spleno- or hepatomegaly can be noted. The broad spectrum of these nonspecific presentations can cause delays in diagnosis, as portrayed in the third case. It is therefore important to be aware of this rare disease and to thoroughly investigate these nonspecific symptoms [1, 5].

Radiological diagnosis remains challenging, as there are no specific characteristics to differentiate PAS from other benign (e.g. hemangioma) or malignant (e.g. littoral cell sarcoma) splenic tumors. On CT-scan lesions can be hypo- or hyperdense and are often heterogeneously contrast-enhancing as in the cases presented here. On MRI, hypo- and hyperintense regions can be found, consistent with hemorrhage or necrosis. When contrast is used, the lesions again appear to be heterogeneous as shown in all three cases, nevertheless these signs can also be compatible with benign hemangiomata [1, 5, 6]. In the third case this led to a delay in diagnosis. We advocate for a good discussion of the clinical presentation with the radiologist to potentially avoid these delays. [^18^F]FDG-PET/CT may show regions of increased metabolism, which is absent in regular benign splenic lesions. The review by Barat et al. reports high sensitivity, specificity, positive predictive value, and negative predictive value of [^18^F]FDG-PET/CT in differentiating benign from malignant solid focal splenic lesions in patients with and without malignant disease (resp. 100%, 100%, 100%, and 100% vs. 100%, 83%, 80%, and 100%) [7]. While literature on PAS diagnosis using [^18^F]FDG-PET/CT is limited, the available reports suggest that FDG-avid splenic lesions are likely to be malignant angiosarcomas [5, 8–10]. An exception is reported by Sözel et al., where splenic lesions showed FDG-avidity, but metastatic liver lesions were not hypermetabolic [11]. In the three cases presented here, all lesions originating from the malignant angiosarcoma in the spleen, liver, and bone were FDG-avid. The first case demonstrated liver lesions which were not FDG-avid. Initially, these were suspected to be malignant, but over time, they were confirmed to be benign hemangiomata as no further progression was observed after treatment with splenectomy only. This indicates that within the imaging modalities, [^18^F]FDG-PET/CT might provide more specificity in differentiating between benign and malignant splenic lesions. However, histopathological confirmation remains the cornerstone for the definite diagnosis of PAS.

**Figure 2:**
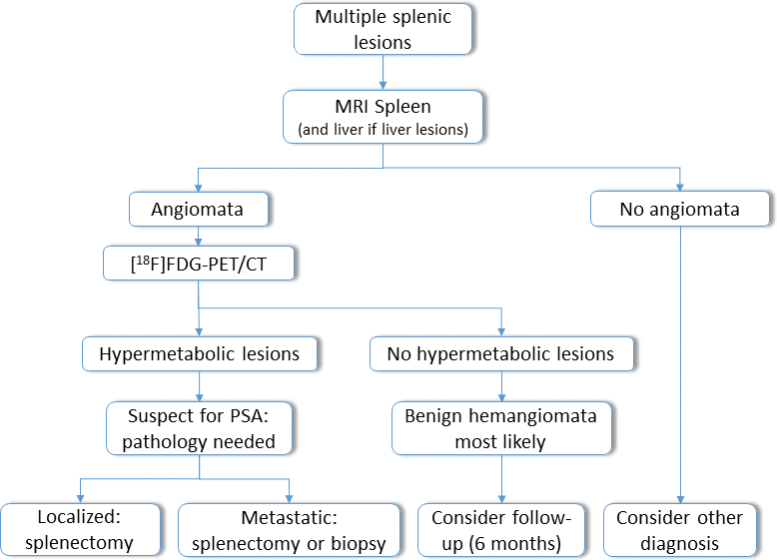
Proposed flowchart of diagnostic algorithm for primary angiosarcoma of the spleen (PAS). MRI: magnetic resonance imaging; [^18^F]FDG-PET/CT: 18-fluorodeoxyglucose-positron emission tomography computed tomography.

A splenic biopsy is generally contra-indicated because of the high seeding risk as well as a considerable risk for hemorrhage or splenic rupture following the biopsy. Therefore, in suspected localized cases, splenectomy is recommended [12]. In our third case, a liver biopsy was performed, because metastatic disease was already suspected on [^18^F]FDG-PET/CT scan. Based on these findings, we propose a flowchart in Figure 2 of a diagnostic algorithm for PAS.

Histopathological characteristic features of PAS include the presence of disorganized anastomosing vascular channels lined with large atypical endothelial cells exhibiting irregular hyperchromatic nuclei. Splenic sinuses may be identifiable, as observed in our third case, where a similar pattern was found in the liver [12]. Immunohistochemical staining is essential and should include vascular proliferation markers (e.g. CD34, CD31, ERG), as performed in the presented cases [12]. Additional staining for human herpes virus 8 is recommended to exclude Kaposi sarcoma. In early stages, mitotic rate and tumor size can be of prognostic value [1]. Only in our third case was the Ki-67-proliferation index elevated, which is consistent with the aggressive nature of PAS in a metastatic setting.

When diagnosed early, the recommended primary treatment for PAS is splenectomy before any potential splenic rupture occurs, as it is the only treatment that benefits OS. It should be noted however, that splenectomy is rarely curative [1]. As for systemic treatment or adjuvant radiation, data from large cohorts are scarce, and no treatment has shown to significantly improve OS.

Systemic therapy is based on chemotherapeutic regimens that are extrapolated from existing sarcoma-regimens with doxorubicin, ifosfamide, and paclitaxel. A phase 2 clinical trial treating metastatic/unresectable soft-tissue angiosarcoma with paclitaxel weekly showed median progression-free survival (mPFS) of 4 months and median OS of 8 months [13]. A retrospective study using gemcitabine as a single agent for angiosarcoma reported a 68% overall response rate (ORR) in 25 patients, with a mPFS of 7 months [14]. In retrospective studies, erubilin has shown activity in angiosarcoma as well, mostly in cutaneous angiosarcoma [15].

Clinical trials with other agents; immune checkpoint inhibitors (ICI), and tyrosine kinase inhibitors (TKI) have been conducted as well. In the largest prospective phase 3 angiosarcoma trial to date pazopanib, a vascular endothelial growth factor receptor (VEGFR) TKI, with insufficient activity as a monotherapy in angiosarcoma, was compared to pazopanib combined with carotuximab, a monoclonal antibody (mAB) binding endoglin which is upregulated in tumor endothelial cells. With a mPFS of 4.3 months compared to 4.2 months respectively, the trial did not meet its primary endpoint [16].

When considering ICI, a case series of patients with breast or cutaneous angiosarcoma showed partial and complete responses in some patients [17].

Currently, there is a phase 2 basket trial investigating the activity of pembrolizumab (anti-PD-1 mAB) in rare cancers. In an interim rapport, 98 patients with rare sarcomas were included, out of which one patient had angiosarcoma that progressed despite treatment [18]. Another phase 2 clinical trial with sintilimab (anti-PD-1 mAB) is being conducted for angiosarcoma (ClinicalTrials.gov ID: NCT05026736).

More recent work shows promising results using the combination of a TKI with chemotherapy and/or ICI. A phase 2 trial with cabozantinib + nivolumab (anti-PD-1 mAB) showed an ORR of 59% and mPFS of 9.6 months in taxane-pretreated angiosarcoma [19]. In the exploratory cohort of a phase II trial combining cabozantinib with temozolomide responses were observed in angiosarcoma [20]. In a pilot study combining lenvatinib with pembrolizumab six angiosarcoma patients had a mPFS of 40.9 weeks, all having stable disease, with one achieving a partial response later on [21].

However, due to the rarity and aggressiveness of soft-tissue sarcomas, placebo-controlled trials are lacking. Initiatives such as the ASC project are of utmost importance. In this project, patients themselves can participate in research by sharing their clinical information and biospecimens, to discover the etiology and potential therapies for this rare entity by pooling data [22]. This helps to overcome the barrier of low prevalence in rare diseases and might provide new insights that could not have been uncovered on a single center level.

Today, the national comprehensive cancer network (NCCN) guidelines recommend paclitaxel, anthracycline-, or gemcitabine-based regimens for the treatment of angiosarcoma [23]. The European society for medical oncology recommends taxanes as the first choice and gemcitabine alone or in combination with docetaxel as an alternative [24]. However, both organizations emphasize the lack of trial data and the need for further research. For PAS specifically, there are no guidelines or trial data available. The second patient presented here was treated with paclitaxel weekly for a metastatic PAS.

In conclusion, primary splenic angiosarcoma is a rare and aggressive neoplasm with challenging diagnostic and management aspects. Awareness for the disease, its nonspecific clinical presentation, and large overlap with benign lesions on various imaging modalities is essential to make a timely diagnosis. Incorporating [^18^F]FDG-PET/CT into the diagnostic algorithm may aid in differentiating between benign and malignant lesions. Whenever possible early splenectomy is recommended. Paclitaxel may be considered as a first-line systemic treatment option. Emerging immunotherapy and tyrosine kinase inhibitor treatment show potential, but more data are needed to evaluate their effectiveness. For now, PAS remains a very aggressive disease for which currently available systemic therapies have only marginal activity.

## Data Availability

The data that support the findings of this study are not publicly available due to their containing information that could compromise the privacy of the research participants.
